# Cerebrospinal fluid estradiol fluctuations during the estrous cycle and their association with intraocular pressure and optic nerve sheath diameter in dogs: a first report

**DOI:** 10.1007/s11259-025-10820-x

**Published:** 2025-07-11

**Authors:** Candemir Özcan, Serkan Bozacı, Berrak Işık Soytürk, Kenan Çağrı Tümer, Ömer Deniz, Tarık Şafak, Ayşe Başak Dellalbaşı, Özgür Kaynar, Elif Dogan

**Affiliations:** 1 Faculty of Veterinary Medicine, Department of Surgery, Kastamonu University, Kastamonu, 37150 Türkiye; 2 Faculty of Veterinary Medicine, Department of Internal Medicine, Kastamonu University, Kastamonu, Türkiye; 3 Faculty of Veterinary Medicine, Department of Obstetrics and Gynecology, Kastamonu University, Kastamonu, Türkiye; 4 Faculty of Veterinary Medicine, Department of Biochemistry, Kastamonu University, Kastamonu, Türkiye

**Keywords:** Optic nerve sheath diameter, Estradiol, Progesteron, Intraocular pressure, Dog

## Abstract

**Objective:**

The relationship between optic nerve sheath diameter (ONSD), an indicator of intracranial pressure (ICP), and intraocular pressure (IOP) is controversial. The two aims of this study were; first, to investigate the effect of cerebrospinal fluid (CSF) estradiol (E2) and progesterone (P4) levels on ONSD and second, to investigate the relationship between ONSD and IOP in female dogs.

**Methods:**

IOP measurements were performed using rebound tonometer, and transpalpebral ultrasonographic assessment of the ONSD was conducted. CSF samples were collected, and E2 and P4 levels were quantified using enzyme-linked immunosorbent assay (ELISA). Multiple regression analysis was conducted to assess the relationships among the variables.

**Results:**

Multiple regression analyses on dogs found that the level of cerebrospinal fluid estradiol level (CSF-E2) significantly influenced left (L) ONSD (*p* = 0.0421, R² = 0.1551) and right (R) ONSD (*p* = 0.0216, R² = 0.1938). Systolic blood pressure (SBP) was a significant independent variable for left intraocular pressure (LIOP) (*p* = 0.0122, R² = 0.2261), while body weight was a significant predictor for right intraocular pressure (RIOP) (*p* = 0.0008, R² = 0.3679).

**Conclusions:**

This study revealed that CSF-E2 levels exert a significant influence on ONSD in dogs, whereas no direct association was observed between IOP and ONSD. These findings underscore the notion that ONSD is modulated not only by hemodynamic mechanisms but also by hormonal regulation.

## Introduction

The optic nerve sheath is a continuation of the dura mater, with the subarachnoid space extending along the optic nerve within this protective sheath. An increase in intracranial pressure (ICP) is transmitted to the optic nerve head, causing swelling of the optic disc and leading to papilledema (Hayreh 1964; Moretti and Pizzi 2009).

The optic nerve sheath diameter (ONSD) is a valuable metric for assessing neurological disorders related to ICP changes and ocular conditions linked to increased intraocular pressure (IOP). The ONSD complex may expand in cases of ICP due to cerebrospinal fluid (CSF) accumulation in the subarachnoid space (Gass et al. 1996; Hansen and Helmke 1997; Lee et al. 2003). Elevated CSF (Ren et al. 2011) and ICP (Sheeran et al. 2000) causes an increase in ophthalmic venous pressure, raising IOP and underlining the link between these pressures, especially in patients with neurologic impairment. However, the relationship between ICP and IOP is still debated in academic discussions, leaving the potential of using IOP as a non-invasive alternative to ICP uncertain (Li et al. 2012).

In addition to these pressure dynamics, there is growing interest in understanding how hormonal fluctuations influence ICP regulation, particularly in female patients. Currently, there is limited understanding regarding the impact of endogenous hormonal status or the presence of synthetic hormones on ICP and post-traumatic brain injury outcomes in females (Maghool and Khaksari 2013). Recent findings indicate that the phase of the estrous cycle in female animals may influence the results of experimental stroke (Roof and Hall 2000).

Hormonal fluctuations, particularly involving sex steroid hormones like E2 and P4, can influence CSF dynamics and ICP regulation. For example, fluctuations during menstrual cycles have been associated with ICP variations (Bhat et al. 2024). Additionally, evidence suggests that progesterone treatment following traumatic brain injury significantly decreases edema, enhances outcomes, and reinstates the integrity of the blood-brain barrier (Herson et al. 2009).

In conditions like idiopathic intracranial hypertension, hormonal imbalances, especially in women, are linked to elevated ICP, with CSF pressure positively correlated with hormone-related changes (Ljubisavljević and Trajković 2016). Sex hormones are also reported to be effective in alleviating brain edema following extensive traumatic brain injury (Ahmadmolai et al. 2008). As elevated ICP leads to brain inflammation, the reduction of ICP by ovarian hormones may, in turn, reduce brain edema (Gilkes and Whitfield 2007).

This study had two aims: first, to examine the effect of CSF E2 and P4 levels on ONSD; second, to investigate the relationship between ONSD and IOP. Since ONSD is often used as an indicator of intracranial pressure (ICP), understanding the relationship between these parameters and IOP may be important for early detection of neurologic conditions. However, direct measurement of ICP was not performed in this study and the relationship between ONSD and IOP was only considered as an indirect indicator.

## Material and method

### Animal

The research included 27 intact mixed-breed female dogs presented to Kastamonu University Animal Hospital for elective ovariohysterectomy. The dogs exhibited a mean age of 2.85 ± 1.46 years (ranging from 1 to 7 years) and a mean body weight of 26.1 ± 8.75 kg (ranging from 15 to 45 kg). All animals have a comprehensive clinical evaluation before the clinical study to confirm their systemic health. Dogs having a history of neurological, ocular, or systemic disorders were eliminated. The study protocol was approved by the Kastamonu University Local Ethics Committee for Animal Experiments (date: 12.11.2024, approval number: 2024/46). All procedures were performed in accordance with the ARVO Statement for the Use of Animals in Ophthalmic and Vision Research.

### Exlusion criteria

Three dogs having optic neuritis and eye surgery were excluded from the research. Hypertension, diabetes, and severe vascular diseases were examined for in dogs, excluding two with hypertension. The remaining dogs were confirmed not to be receiving medications that affect cerebrospinal fluid dynamics or intraocular pressure, including corticosteroids or diuretics. Hormonal factors were taken into account, leading to the exclusion of dogs undergoing hormone replacement therapy, estrus suppressor medications, or those that were pregnant or lactating. Furthermore, dogs with acute or chronic infections, autoimmune diseases, or a recent history of head trauma were excluded to mitigate potential confounding effects on the results.

### Intraocular pressure measurments

The intraocular pressure (IOP) was assessed using a rebound tonometer (TonoVet, iCare, Finland), which is extensively validated for application in canine patients owing to its precision and non-invasive characteristics. Measurements were conducted on both the right and left eyes, with three consecutive readings collected from each eye to improve measurement reliability. The order of intraocular pressure measurement between right and left eyes was randomized for each subject to prevent order-related bias. The final intraocular pressure (IOP) for each eye was determined by measuring the average of the three readings obtained. A constant working distance (~ 5 mm) and perpendicular alignment to the cornea were maintained in accordance with the manufacturer’s guidelines, using visual confirmation and the alignment markers provided on the tonometer.

Following to the IOP assessment, a thorough examination of the eyes was conducted to identify any indications of trauma or corneal irritation. Measurements were discarded if intra-session variability exceeded 5 mmHg or if the instrument displayed an error. Corneal integrity was assessed using fluorescein staining after measurement.

All measurements were performed by a single experienced clinician to reduce inter-observer variability. The IOP values were subsequently analyzed alongside CSF hormone levels and optic nerve sheath diameter (ONSD) measurements to evaluate potential correlations between intraocular and intracranial pressure dynamics.

### Cerebrospinal fluid collection and patient monitoring

Upon admission to Kastamonu University Animal Hospital, all dogs received a thorough clinical examination, which included a physical assessment, body temperature measurement, and cardiovascular evaluation. Blood samples were obtained for standard hematological and biochemical analyses to assess systemic health and exclude any underlying conditions that may affect the results. Blood pressure was measured non-invasively using a patient monitor (Comen C80-V, Shenzhen, China) device with a cuff placed on the forelimb. Measurements were obtained within 10 min of IOP evaluation to minimize variability due to stress.

Cerebrospinal fluid (CSF) samples were collected under general anesthesia. Following anesthetic induction with Xylazine (Rompun^®^ %2, 2.2 mg/kg intramuscular), the dogs were positioned in lateral recumbency, and the atlanto-occipital region was prepared aseptically. A 22-gauge spinal needle was introduced into the cisterna magna through the atlanto-occipital space. The needle was carefully advanced until cerebrospinal fluid (CSF) flow was observed and 2 mL of CSF was collected in a sterile polypropylene tube.

The samples were immediately placed on ice and stored at −80 °C until analysis using enzyme-linked immunosorbent assay (ELISA). Measures were implemented to prevent blood contamination, and samples exhibiting visible blood traces were excluded from analysis. The dogs were monitored during recovery to identify potential complications, including respiratory depression or neurological signs.

### Ultrasound measurement of the optic nerve sheath diameter (US-ONSD)

Optic nerve sheath diameter was measured an with ultrasound (Versana Active, General Electric) using a microconvex transducer (8 C-RS, General Electric) with transorbital approach.

### Statistical analysis

Data analysis was conducted utilizing SPSS (IBM SPSS Statistics for Windows, Version 26.0) software. Descriptive statistics, including mean, standard deviation, minimum, and maximum values, were computed for the demographic and biological data of the dogs in the study. The data obtained are presented as mean ± standard deviation. The normality of the data was assessed using the Shapiro-Wilk test. The test results indicate that R-ONSD, L-ONSD, CSF-E2, cerebrospinal fluid P4 level (CSF-P4), heart rate, rectal body temperature, RIOP, LIOP, and diastolic pressure data exhibited a normal distribution (*p* > 0.05). Age (*p* < 0.001) and body weight (*p* = 0.024) exhibited non-normal distribution. Consequently, nonparametric tests were utilized for these two variables. Parametric analyses were conducted for the remaining data. Multiple regression analysis was conducted to assess the relationships among the variables and to evaluate the impact of the independent variables on the dependent variables. Coefficient of determination (R^2^) value was computed to assess the explanatory power of each model. The significance level was established at *p* < 0.05.

## Results

The study included 27 intact mixed-breed female dogs with a mean age of 2.85 ± 1.46 years (ranging from 1 to 7 years) and a mean body weight of 26.1 ± 8.75 kg (ranging from 15 to 45 kg). The mean rectal body temperature was 39.2 ± 0.61 °C (range: 38.2–40.2 °C), heart rate was 107 ± 28.2 beats per minute (bpm) (range: 36–158 bpm), systolic blood pressure was 152 ± 27.4 mmHg (range: 93–240 mmHg), and diastolic blood pressure was 93.1 ± 17.4 mmHg (range: 54–135 mmHg).

**Fig. 1:**
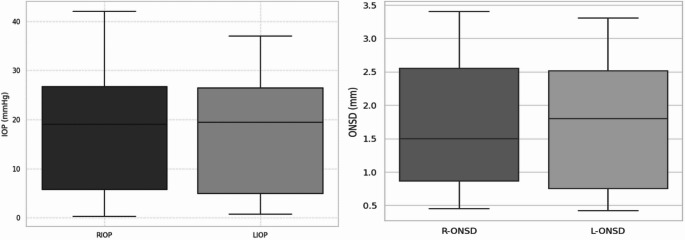
Distribution of right and left intraocular pressure (IOP) and optic nerve sheath diameter (ONSD) values measured in dogs

**Fig. 2:**
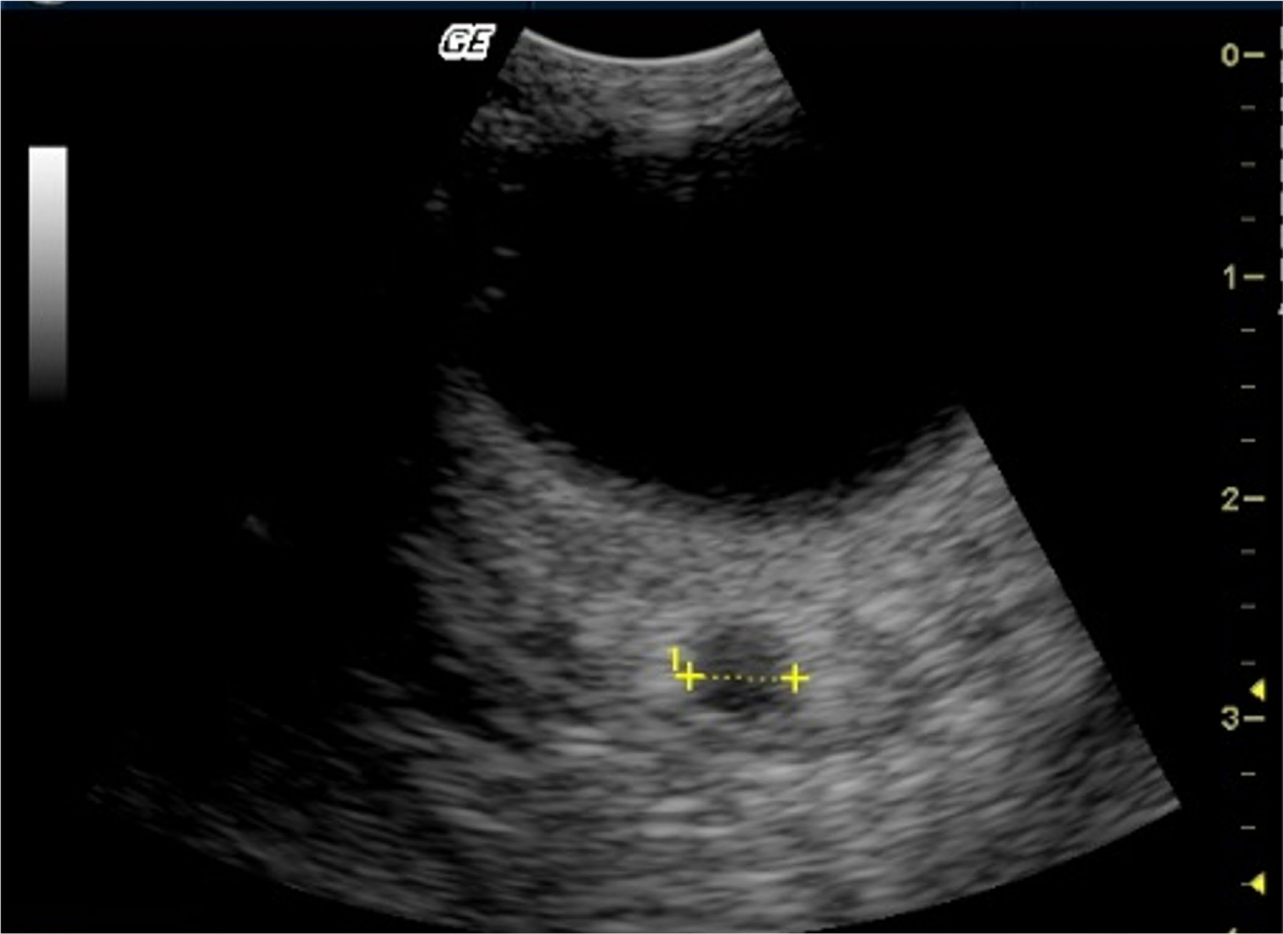
The figure represents the upper transorbital ultrasound image of dogs. All measurements were obtained between the points indicated by two crosshairs connected by a dotted line. R-ONSD ranged from 1.50 mm to 3.40 mm. L-ONSD ranges from 1.80 mm to 3.30 mm

The mean optic nerve sheath diameter (ONSD) and intraocular pressure (IOP) measured in the right eye were 2.5 ± 0.45 mm (range: 1.50–3.40 mm) and 26.6 ± 7.33 mmHg (range: 14–42 mmHg), respectively. In the left eye, the mean ONSD was 2.53 ± 0.42 mm (range: 1.80–3.30 mm), and the IOP was 26.3 ± 6.29 mmHg (range: 13–37 mmHg). Figure 1 illustrates the comparative distribution of the right and left measurements using boxplots. Transorbital ONSD ultrasound measurement is shown in Fig. 2.

The cerebrospinal fluid (CSF) estradiol (E2) level was 130 ± 19.5 pg/ml, and the progesterone (P4) level was 12.1 ± 2.68 ng/ml. Descriptive statistics for the variables measured in the included dogs are presented in Table 1.

**Table 1:** Descriptive statistics of all variables

	*R*-ONSD	L-ONSD	CSF-E2	CSF-P4	Age	Body Weight	Hearth Rate	Rectal Body Temperature	RIOP	LIOP	Systolic Pressure	Diastolic Pressure
N	27	27	27	27	27	27	27	27	27	27	27	27
Mean	2.50	2.53	130	12.1	2.85	26.1	107	39.2	26.6	26.3	152	93.1
Median	2.60	2.50	128	11.6	2	24	100	39.2	24	26	150	94
Std. Dev.	0.45	0.42	19.5	2.68	1.46	8.75	28.2	0.61	7.33	6.29	27.4	17.4
Minimum	1.50	1.80	87	7.75	1	15	36	38.2	14.	13	93	54
Maximum	3.40	3.30	171	18.7	7	45	158	40.2	42	37	240	135
Shapiro- Wilk W	0.975	0.968	0.989	0.964	0.815	0.911	0.981	0.936	0.956	0.973	0.924	0.977
Shapiro-Wilk p	0.748	0.541	0.991	0.461	< 0.001	0.024	0.884	0.098	0.294	0.696	0.049	0.788

The multiple regression analysis performed on the left (L) optic nerve sheath diameter (ONSD) revealed that cerebrospinal fluid estradiol (CSF-E2) levels made a statistically significant contribution to the model (*p* = 0.0421). The model explained 15.51% of the total variance (R² = 0.1551). In contrast, regression analysis evaluating the effect of left intraocular pressure (LIOP) on L-ONSD showed that the independent variable did not contribute significantly to the model (*p* = 0.9084), and the model’s explanatory power was extremely low (R² = 0.0004). Similarly, the regression analysis assessing the effect of CSF progesterone (CSF-P4) on L-ONSD indicated no significant contribution of the independent variable to the model (*p* = 0.2510), with a low explanatory power (R² = 0.0524).

**Fig. 3:**
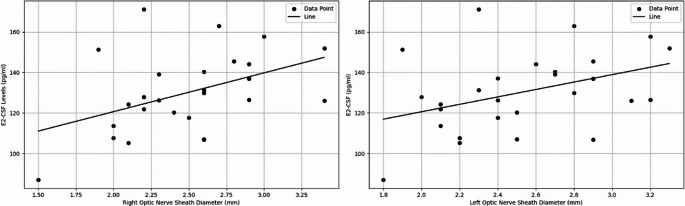
Regression graph showing the relationship between ONSD and CSF-E2 in dogs

The effect of cerebrospinal fluid estradiol (CSF-E2) on the right (R) optic nerve sheath diameter (ONSD) was statistically significant (*p* = 0.0216), and the model demonstrated a moderate explanatory power (R² = 0.1938). The effect of cerebrospinal fluid progesterone (CSF-P4) on R-ONSD was not statistically significant (*p* = 0.0813), and the model had a low explanatory power (R² = 0.1166). Similarly, the effect of right intraocular pressure (RIOP) on R-ONSD was not statistically significant (*p* = 0.3478), with the model exhibiting very low explanatory power (R² = 0.02759). Figure 3 illustrates the relationship between ONSD and CSF-E2 in dogs.

In the multiple regression analysis for left intraocular pressure (LIOP), systolic blood pressure (SBP) was identified as a significant independent variable, with the model explaining 22.61% of the total variance (*p* = 0.0122, R² = 0.2261). The regression plot is presented in Fig. 3. In the multiple regression analysis for right intraocular pressure (RIOP), SBP also showed a statistically significant effect (*p* = 0.050); however, the model accounted for only 8.29% of the total variance (R² = 0.0829), indicating a rather weak explanatory power for this relationship.

**Fig. 4:**
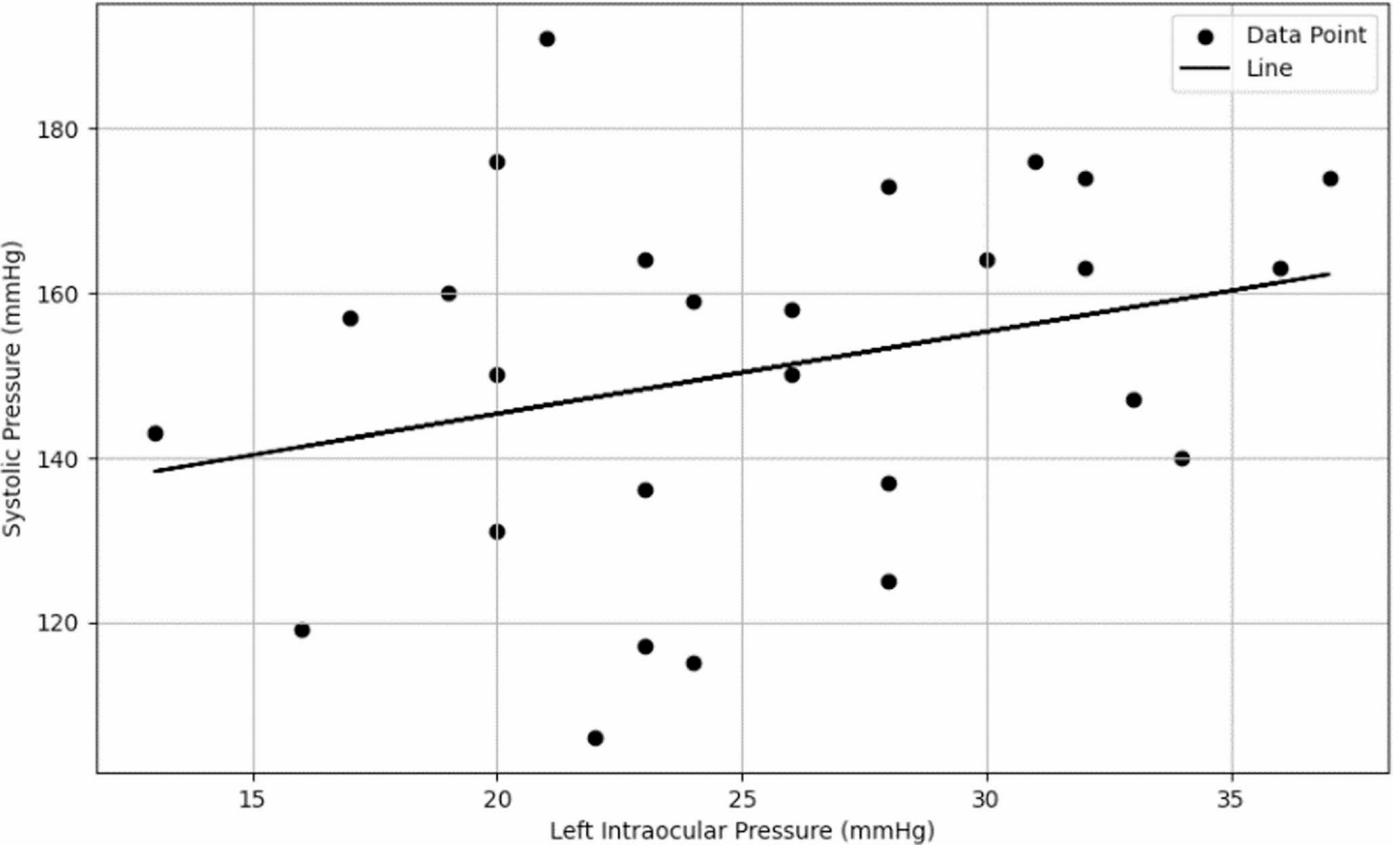
Regression graph showing the relationship between LIOP and SBP in dogs

The regression analysis evaluating the effect of CSF-P4 on left intraocular pressure (LIOP) revealed that the independent variable did not make a significant contribution to the model (*p* = 0.9540). The model demonstrated very low explanatory power (R² = 0.0001). Similarly, the regression analysis assessing the effect of CSF-E2 on LIOP showed no significant contribution from the independent variable (*p* = 0.4823), with low explanatory power (R² = 0.0199). The effect of CSF-P4 on right intraocular pressure (RIOP) was also found to be statistically non-significant (*p* = 0.5855), and the model exhibited very low explanatory strength (R² = 0.01159). Furthermore, the effect of CSF-E2 on RIOP was not statistically significant (*p* = 0.5249), and the model demonstrated minimal explanatory power (R² = 0.01636). Figure 4 illustrates the relationship between LIOP and SBP in dogs.

**Fig. 5:**
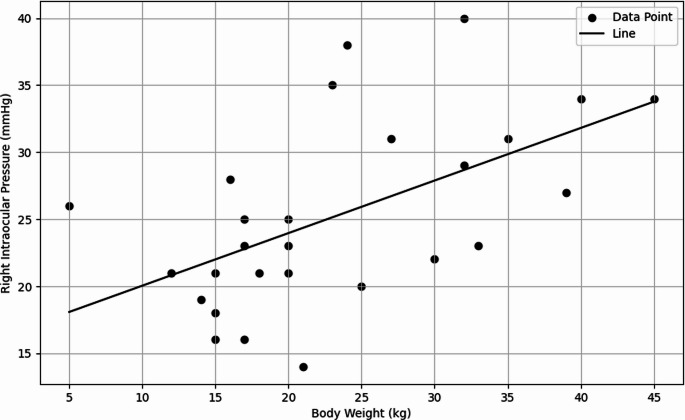
Regression graph showing the relationship between RIOP and body weight in dogs

The multiple regression analysis evaluating the effect of body weight on right intraocular pressure (RIOP) demonstrated that body weight made a statistically significant contribution to the model (*p* = 0.0008). The model explained 36.79% of the total variance (R² = 0.3679). Although body weight was also a significant predictor of left intraocular pressure (LIOP), the explanatory power of the model was found to be low (R² = 0.1670). Figure 5 illustrates the relationship between RIOP and body weight in dogs.

## Discussion

In the present study, CSF E2 and P4 levels were measured in female dogs without any neurologic disorders or diseases. IOP measurements were also performed in these dogs. The aim of the study was to evaluate the correlations between CSF E2 and P4 levels, as well as IOP measurements, with ONSD.

Neuroactive steroids play critical roles in neurophysiological and behavioral processes (Martin et al. 2019). Steroid hormones such as progesterone (P4), testosterone, and estradiol (E2) are synthesized from cholesterol in classical steroidogenic peripheral tissues such as the adrenal glands, gonads, and placenta, and exert various physiological effects. These hormones are capable of crossing the blood-brain barrier and reaching brain tissues, thereby modulating neuronal functions at the central level (Hobbs et al. 1992; Tsutsui et al. 2000). It has been demonstrated that these steroids can undergo further metabolism within the brain, and that the brain itself is capable of synthesizing steroids from cholesterol (Porcu et al. 2016; Tsutsui et al. 1999). Steroids synthesized within the brain are referred to as neurosteroids, and their roles in the regulation of brain function are gaining increasing importance (Tsutsui et al. 2000).

In this study, multiple regression analysis on L-ONSD revealed that CSF-E2 levels made a significant contribution to the model (*p* = 0.0421). This finding highlights the potential effect of E2 on ONSD and provides important evidence regarding the interaction of neuroactive steroids with brain physiology. The model explained 15.51% of the total variability (R² = 0.1551), indicating that the effect of CSF-E2 on L-ONSD is measurable but limited. Similarly, the effect of CSF-E2 on R-ONSD was also statistically significant (*p* = 0.0216), and the explanatory power of the model was found to be moderate (R² = 0.1938). These results suggest that E2 may have a specific influence on ONSD.

The local synthesis of E2 in the brain plays a crucial role in the regulation of brain function and behavior under physiological conditions. It has also been noted that E2 exhibits homeostatic effects in the brain (Srivastava et al. 2013). Endogenous or exogenous estrogen exposure contributes to the dilation of cerebral arteries, resulting in changes in cerebral blood flow. This effect is mediated by an increase in the production of vasodilator factors such as nitric oxide and prostacyclin (Duckles and Krause 2007). In women, elevated estrogen levels during the menstrual cycle and pregnancy lead to changes in cerebral blood flow (Brackley et al. 1999). Numerous studies have shown that cerebral blood flow is directly proportional to ONSD and ICP (Kim et al. 2014; Kondrashova et al. 2019). In our study, the significant correlation found between ONSD measurements and CSF-E2 levels provides valuable insights into the effects of this hormone on the central nervous system. Specifically, the significant effect of CSF-E2 levels on both left and right ONSD (L-ONSD: *p* = 0.0421, R² = 0.1551; R-ONSD: *p* = 0.0216, R² = 0.1938) supports the relationship between estrogen and cerebral circulation, as well as intracranial pressure indicators. These findings align with previous research suggesting that estrogen may increase cerebral blood flow through its vasodilatory effects, leading to expansion of the subarachnoid space and resulting in an increase in ONSD (Duckles and Krause 2007; Kim et al. 2014).

Pregnancy has been shown to cause changes in IOP in cats through hormonal regulation, as supported by our previous findings (Ozcan et al. 2024). In the current study, significant effects of neurosteroid hormone levels, particularly E2, in the CSF on ONSD were observed. However, CSF-E2 and CSF-P4 levels did not have a significant effect on IOP (*p* > 0.05). Similarly, the effect of CSF-P4 on ONSD was not statistically significant. These findings highlight that systemic or central mechanisms cannot be evaluated through a single marker and emphasize the importance of considering both CSF hormone levels and systemic hemodynamic parameters (e.g., SBP) together.

Additionally, the lack of a significant effect of P4 on ONSD in the study highlights the more pronounced effect of E2 in this context. This finding may support the hypothesis that estrogen plays a dominant regulatory role in certain physiological processes. However, it should also be considered that hormonal effects can become complex due to individual variations, receptor sensitivity, and interactions within hormonal signaling pathways. In this study, the regression analysis evaluating the effect of CSF-P4 on ONSD showed that this hormone did not make a significant contribution to the model. While estrogen’s anti-inflammatory effects mitigate ischemic brain damage, this vasoprotective benefit decreases in the presence of progestagens, indicating an antagonistic relationship between estrogen and progesterone. However, it is also suggested that, especially in physiological processes such as reproductive health, cerebrovascular functions, and bone health, they may work synergistically (Sunday et al. 2006). In this context, the lack of a significant effect of progesterone on ONSD in our study suggests that this hormone does not induce a notable change in cerebral vascular structures directly.

The optic nerve is a robust protrusion of brain tissue fully encapsulated by the dura, arachnoid, and pia mater, and its size varies in response to CSF transmission and changes in ICP (Helmke and Hansen 1996; Moretti and Pizzi 2009). In this context, the use of ONSD and IOP as non-invasive indicators in the exclusion of ICP is of great importance; however, studies have shown that methods such as ONSD and IOP do not always yield consistent results (Li et al. 2012; Li-min et al. 2018). While some studies suggest a significant correlation between ICP and IOP (Ren et al. 2011; Sheeran et al. 2000), it has been reported that in patients with primary open-angle glaucoma, the reduction of CSF pressure around the optic nerve and the subsequent narrowing of ONSD, despite normal IOP, may play a role in the development of glaucoma (Wang et al. 2012).

Moreover, in our study, the different levels of neurosteroid hormones (E2 and P4) in the CSF complicate the situation. In our regression analysis, no significant relationship was observed between LIOP and L-ONSD (*p* = 0.9084, R² = 0.0004), and the effect of RIOP on R-ONSD was also not statistically significant (*p* = 0.3478, R² = 0.02759). These findings suggest that the relationship between ONSD and IOP alone is not decisive in patient evaluation, and other factors such as neurosteroid hormones and body weight contribute to the complexity of this interaction. In a few cases, particularly one dog with IOP values of 42 mmHg (right eye) and 37 mmHg (left eye), elevated readings may reflect acute stress responses during measurement. Although stress-minimizing protocols were applied, individual variability in stress sensitivity may have influenced IOP and potentially CSF hormone levels.

The existence of a relationship between body weight and IOP is expressed in several studies. It is emphasized that increased body weight is a risk factor for glaucoma and thus for IOP elevation. For example, as the prevalence of obesity increases, the risk of high IOP has also been observed to rise (Chen et al. 2021; Stojanov et al. 2013). This relationship is thought to be due to multiple physiological mechanisms, including changes in ocular blood flow and the pressure exerted by increased retrobulbar adipose tissue (Stojanov et al. 2013; Xu et al. 2012). It is also stated that advancing age, along with high body mass index (BMI), significantly increases the risk of glaucoma (Nirmala et al. 2014). In our study, the body weight variable was found to complicate the relationship between ONSD and IOP in dogs. The multiple regression analysis evaluating the effect of body weight on RIOP showed that the model explained 36.79% of the total variability (*p* = 0.0008). It is known that obesity and obesity-related systemic diseases pose a greater risk for blinding eye diseases such as cataracts, glaucoma, and age-related macular degeneration (Cheung and Wong 2007). Stojanov et al. (2013) emphasize that IOP elevation in obese individuals is due to changes in ocular blood flow caused by physical pressure. The findings in the dogs included in our study were consistent with the aforementioned literature. Therefore, body weight should be considered a potential confounding factor in the interpretation of the relationship between ONSD and IOP in dogs.

SBP causes changes in ocular perfusion, leading to changes in mean ocular perfusion pressure. These changes are directly influenced by IOP and blood pressure (Ren et al. 2011). It is known that patients with systemic hypertension exhibit higher IOP compared to normotensive individuals. This suggests that high blood pressure may contribute to mechanisms that increase IOP (Parajuli et al. 2021). It has been proposed that an increase in SBP may raise the risk of developing primary open-angle glaucoma and ocular hypertension (Kaskar et al. 2022). In this context, multiple regression analyses conducted in our study to evaluate the relationship between SBP and IOP found that SBP had a significant effect on LIOP (*p* = 0.0122, R² = 0.2261). This finding highlights the determining effect of systemic blood pressure on IOP and is consistent with the data in the literature. Similarly, in the analysis of RIOP, a significant effect of SBP was observed (*p* = 0.05, R² = 0.0829); however, the low explanatory power of the model suggests that this relationship may be weak but statistically borderline significant. Especially in hypertensive dogs, it should be considered that an increase in SBP could affect the physiology of ocular structures and cause an increase in IOP levels. Furthermore, this discrepancy, despite the randomisation of the measurement sequence, can be explained by individual variability, small asymmetries in the measurement conditions or stress-related physiological differences between the eyes.

The research on ICP suggests that it interacts with both IOP and systemic arterial pressure, indicating a systemic mechanism that could influence all three variables simultaneously (Ren et al. 2011). These findings support the idea that IOP and SBP should be considered as factors in the pathophysiology of ICP. Our study highlights the complexity of the relationships between IOP, ONSD, and SBP, and reinforces the notion that these variables should not be viewed as independent but as parts of an integrated system.

This study has some limitations. First, the sample size (female dogs) is relatively small, which may limit the generalizability of the results to the broader population. Additionally, since this research was conducted only on healthy female dogs, different results may be obtained in individuals with neurological disorders. The measurement of CSF E2 and P4 levels was only done at a single time point, meaning that changes in hormone levels over time were not considered. Furthermore, ICP was not measured invasively in this study, which prevents a deeper understanding of the relationship between ONSD and IOP. Although the order of eye measurements was randomized, a greater stress response during the initial eye examination—regardless of whether it involved the left or right eye—cannot be entirely ruled out. This acute stress reaction may have influenced physiological parameters such as SBP and IOP, potentially contributing to asymmetric relationships between eyes and should be considered in future studies. Moreover, as IOP measurements and ONSD evaluations reflect a single observation, more temporal data may be required to account for individual variations. Although systemic factors such as body weight and SBP were considered, other potential influencing parameters (e.g., genetic factors or nutritional status) were not addressed, which makes it necessary to explore how these factors may affect the results. Future studies with larger sample sizes and long-term follow-up are essential to overcome these limitations and validate the findings.

In conclusion, it was shown that CSF-E2 levels had significant effects on both left and right ONSD. However, no direct relationship was found between IOP and ONSD. It was observed that IOP is more influenced by systemic factors, particularly parameters such as SBP and body weight. Additionally, while CSF-E2 levels had a pronounced effect on ONSD, no significant effect of CSF-P4 levels on ONSD was found. These findings highlight that ONSD is influenced not only by hemodynamic and mechanical factors but also by hormonal regulation, emphasizing that these variables should be evaluated as part of an integrated system, rather than independently.

## Data Availability

No datasets were generated or analysed during the current study.
